# Social robots in a translanguaging pedagogy: A review to identify opportunities for robot-assisted (language) learning

**DOI:** 10.3389/frobt.2022.958624

**Published:** 2022-10-14

**Authors:** Rianne van den Berghe

**Affiliations:** Windesheim Flevoland, Almere, Netherlands

**Keywords:** social robots, human-robot interaction, language learning, translanguaging, educational robots

## Abstract

This mini review discusses the use of social robots in a translanguaging pedagogy: the use of robots to enable students to use their full linguistic repertoire within schools, so any language that they speak at home or in another aspect of their lives. Current research on robot-assisted second-language learning is reviewed with the aim of finding out whether students’ languages have been employed strategically to support learning of another language. A total of 83 articles has been analyzed on the use of first and second languages in student-robot interactions. Most interactions were either exclusively in the second language, or exclusively in the first language, with only target words in the second language. Few studies strategically mixed the two languages to bootstrap learning, and only one study used the first language of students with migrant backgrounds to learn the second language. The review concludes with recommendations for future use of social robots in a translanguaging pedagogy.

## Introduction

Social robots are useful tools for language education. Their physical and social presence (i.e., embodiment) is an important advantage over other types of technology, such as tablets and computers. This could be a main reason for why social robots are found to be more effective than other types of technology in education in general (Belpaeme et al., 2018) and language education in particular (Lee and Lee, 2022). Robots’ physical and social presence is thought to be more motivating and to elicit more social behavior from interaction partners compared to their virtual counterparts. Moreover, social robots, many of which have a humanoid or animal-like body, often have arms, which allows them to make gestures. Gestures are particularly relevant for language learning, as (human) gestures help convey the meaning of language (e.g., through depicting a word’s meaning, in case of iconic gestures) and thus provide visual support (Rowe et al., 2013; Rohlfing, 2019). A recent review on the use of gestures in robot-assisted (language) learning [RA(L)L] has shown that robot gestures benefit interactions and learning as well (de Wit et al., 2022).

One ability of social robots has been discussed less often: their ability to speak any language. Of course, robots share this ability with other types of technology, which is perhaps why it has received less attention. However, it is particularly relevant in light of a pedagogical approach that has gained momentum over the last couple of years: translanguaging. Translanguaging is the use of students’ full linguistic repertoire within schools (García, 2009). In practice, this means that other languages that students speak beside the school language are positively valued and actively used in communication and learning within the school. For a long time, it was believed that languages should be separated (as mixing languages was thought to confuse multilingual students) and that multilingual students should be immersed in the school language to best learn that language. However, language mixing does not confuse students (de Houwer, 1990; Petitto et al., 2001; Meisel, 2010) and immersion programs are not better than bilingual programs for multilingual students’ language development or wellbeing (Sierens and van Avermaet, 2014). The use of other languages within the school actually supports students in learning the school language (García and Li, 2015; García and Lin, 2017; Günther-van der Meij et al., 2020), in learning other subjects, and in their wellbeing in the school (Hornberger, 2005; Cummins 2008; Creese and Blackledge, 2010; Cummins 2019).

There are dozens of activities using students’ first language (L1) which can be done within schools to support students’ learning. Such activities include: focusing on similarities and differences between the L1 and second language (L2), allowing students to discuss assignments amongst each other in the L1, multilingual label quests (eliciting vocabulary items from students in multiple languages), use of multiple languages in book reading or writing, pre-teaching in the L1 (at home or at school), cognate comparison (comparing similar words in different languages), and target word explanations in the L1 (Ticheloven et al., 2020; Bosma et al., 2022). An issue in translanguaging is that teachers may not know how to engage students in multilingual activities or may be afraid to ‘lose control’ by allowing students to speak other languages. Teachers often do not speak students’ L1s, which leads to difficulties in using these languages in the classroom. This presents unique opportunities to use social robots (and other types of technology) in classroom, as robots are able to speak any language.

The field of (educational) robots has potential to promote inclusiveness (Daniela and Lytras, 2019) and the idea of using social robots as a multilingual agent to mediate between speakers of different language backgrounds is not completely novel. Kim (2016) advocated for social robots as ‘cultural brokers’ to mediate between children of different cultural backgrounds. Multilingual robots or tangibles have been developed for migrant populations in the fields of both healthcare and education (Özcan et al., 2014; Kim et al., 2021) and even to encourage the active use of endangered languages (Taylor et al., 2020). For example, a bilingual robot speaking both Spanish and English was used in the study by Kim et al. (2021) to stimulate positive interactions between children with different language backgrounds. Such efforts are worthwhile to positively engage children of different backgrounds in education, or to ensure that people communicate effectively in healthcare. The focus of the current review is on the educational use of robots, as the main goal of translanguaging is to support learning.

The research question of the current review is how languages have been used so far in RALL research and whether there are opportunities for robots in a translanguaging pedagogy to support multilingual students. Existing reviews on RALL (e.g., Randall, 2019; van den Berghe et al., 2019; Lee and Lee, 2022) have focused on several aspects of robots and how they affect learning. For example, Randall (2019) describes how the form, voice, immediacy, non-verbal cues, and personalization of the robot affect learning outcomes and motivation in RALL. Lee and Lee (2022) have conducted a meta-analysis of 16 RALL studies and found a medium effect size (d = 0.59, SE = 0.09) of RALL over non-RALL conditions. They investigated the effects of several moderator variables (age group, target language, language domain, robots’ role, interaction type, and type of non-RALL condition). Although some differences in learning outcomes appeared, they were not statistically significant. How exactly languages have been employed and whether this has been done strategically to support students’ learning, is still an open question. The outcomes of the current review will help in making informed choices when designing robots as tools for (language) education in a translanguaging pedagogy.

## Methods

**FIGURE 1:**
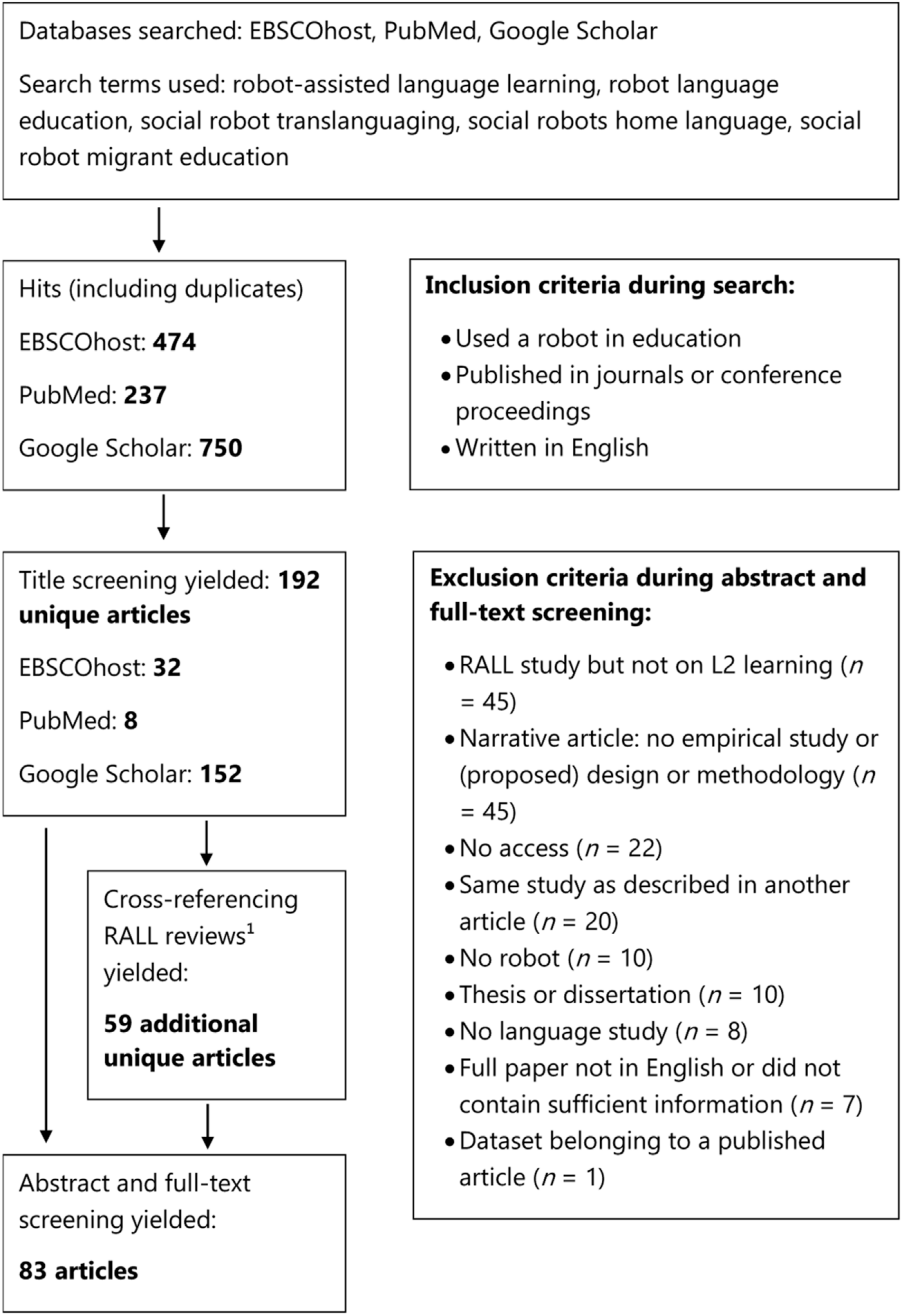
Article selection process.

I conducted a literature search on Google Scholar, EBSCOhost and PubMed using the search terms “robot assisted language learning”, “robot language education”, “social robots translanguaging”, “social robots home language” and “social robot migrant education”. For Google Scholar, a maximum of 150 articles was screened for each search term (cf. Shultz, 2007; Falagas et al., 2008). The main aim of this search was to find studies that used educational robots and included multilingual students’ L1 (both in language education and education in general), or were aimed at teaching an L2. The focus lied on the robot activities rather than on the experimental outcomes of the studies, and, as a result, the exclusion criteria were not very strict in terms of research design. Other reviews (e.g., van den Berghe et al., 2019; Lee and Lee, 2022) had stricter exclusion criteria as they focused on the outcomes of RALL, which is why the current review contains more studies than earlier reviews. Figure 1 shows the complete selection process, including exclusion criteria.^1^ For example, any RALL studies in which monolingual children were taught new words in their L1 (e.g., Tolksdorf et al., 2021) were excluded, because the goal was to study how languages were used in a multilingual setting. Each study was coded on their use of languages on a scale of ‘completely L1, only target vocabulary in L2’ to ‘full L2 program’.

## Results

Table 1 summarizes the 83 articles and their usage of L1 and L2. Many studies (*n* = 24, the upper two rows of Table 1) mainly used the L1 during the student-robot interactions, with only the target words (and perhaps some support words or sentences) in the L2. This approach was used particularly often for novice learners, such as young children who do not have prior knowledge of the L2. The use of the L1 in these studies is therefore not prompted by a desire for translanguaging, but from a practical perspective, as students do not have enough prior knowledge to engage in interactions with more L2. The participant groups of these studies were homogenous in their languages; their L1 is the same as the school language, and the L2 is a foreign language. So, even though there is usage of L1 in these 24 studies, they are not examples of translanguaging in the sense that languages spoken outside of the school were used.

**TABLE 1:** Overview of usage of L1 and L2 in RALL studies.

Amount of L1/L2	Studies	Example of activities
Student-robot interaction completely in L1; target vocabulary (or structures) in L2 (*n* = 22)	Saerbeck et al. (2010); Tanaka and Matsuzoe (2012); Mazzoni and Benvenuti (2015); Herberg et al. (2015); Perlmutter et al. (2016); Kennedy et al. (2016); Schodde et al. (2017); Kanero et al. (2018a); van den Berghe et al. (2018); de Wit et al. (2018); Schodde et al. (2019); Keane et al. (2019); Verhagen et al. (2019); van Minkelen et al. (2020); Demir-Lira et al. (2020); de Haas and Conijn (2020); de Haas et al. (2020); de Wit et al. (2020); de Haas et al. (2021); Molenaar et al. (2021); Kanero et al. (2021); Kanero et al. (2022)	“I spy with my little eye” game in L1, in which target words are offered in L2
Student-robot interaction mostly in L1; target vocabulary (or structures) + support or carrier words provided in L2 (*n* = 2)	Vogt et al. (2019); Chew and Chua (2020)	Vocabulary lesson in which L2 target vocabulary was embedded in L2 sentences
Mix of L1 and L2 in student-robot interaction (*n* = 13)	Alemi et al. (2014); Alemi et al. (2016); Alemi et al. (2017); Alemi and Basirib (2016); Alemi and Haeri (2020); Alemi and Bahramipour (2019); Chang et al. (2010); Gordon et al. (2016); Kory Westlund et al. (2015); Leeuwestein et al. (2021); Thinh et al. (2020); Tseng and Paseki (2022); Wu et al. (2015)	A robot as teacher assistant speaking L2, while the teacher used L1
Student-robot interaction mostly in L2; some support in L1 (*n* = 2)	You et al. (2006); Tanaka et al. (2015)	A robot mostly speaking L2, but also translating target words to L1
Student-robot interaction completely in L2 (*n* = 44)	Arar et al. (2021); Banaeian and Gilanlioglu (2021); Chen et al. (2011); Chen Hsieh and Lee (2021); Cumbal (2020); Engwall and Lopes (2020); Engwall et al. (2021); Funakoshi et al. (2011); Goossens et al. (2019); Halbach et al. (2021); Han (2012); Hemminki and Erkinheimo-Kyllönen (2017); Hong et al. (2016); Hur and Han (2009); In and Han (2015); Kanda et al. (2004); Khalifa et al. (2017); Khalifa et al. (2018); Kim et al. (2014); Konijn et al. (2022); Kory Westlund et al. (2017); Kouri et al. (2020); Kwon et al. (2010); Lee et al. (2010); Iio et al. (2019); Lopes et al. (2017); Lu et al. (2007); Moussalli and Cardoso (2016); Mubin et al. (2013); Najima et al. (2021); Nakamura et al. (2015); Park et al. (2011); Park et al. (2019); Rosenthal-von der Pütten et al. (2016); Schulz et al. (2020); Shin and Shin (2015); Tanaka et al. (2013); Tanaka et al. (2014); Tsai (2019); Vogt et al. (2017); Wang and Johnson (2016); Wang et al. (2009); Wang et al. (2013); Wu et al. (2019)	Conversation classes in L2

Even more studies (*n* = 46, the lower two rows of Table 1) used (almost) exclusively the L2 in the student-robot interaction: an immersion approach. Most studies used the robot to engage in conversations with students in the L2 or to read stories in the L2 to students. The rationale for using such an immersion approach is that the robot provides L2 input of high quality to students who may not have access to native speakers, or that speaking to a robot is less anxiety-inducing than speaking to classmates or other people. This latter has been confirmed in RALL research (Wang et al., 2013; Alemi et al., 2015; Alemi et al., 2017).

Surprisingly few articles (*n* = 13) have adopted an approach in which the L1 and L2 were mixed. Some studies used a ‘one person/character, one language’ approach, in which the robot used one language, and the teacher used the other language (Chang et al., 2010; Alemi et al., 2014; Alemi and Basirib, 2016; Alemi et al., 2016; Alemi et al., 2017; Alemi and Bahramipour, 2019; Alemi and Haeri, 2020). A similar approach is used by Gordon et al. (2016) and Kory Westlund et al. (2015), although the robot mainly used the L1 and a virtual character on a screen spoke both the L1 and L2. In only a few studies, the actual robot was multilingual and used the L1 to support learning of the L2 (Thinh et al., 2020; Leeuwestein et al., 2021; Tseng and Paseki, 2022). Thus, the advantage of the robot being able to easily switch between languages, has not been used very often so far.

Sixteen of the 83 studies (partly) targeted a migrant population (Kim et al., 2014; Gordon et al., 2016; Rosenthal-von der Pütten et al., 2016; Kory Westlund et al., 2017; Lopes et al., 2017; Hemminki and Erkinheimo-Kyllönen, 2017; Goossens et al., 2019; Park et al., 2019; Engwall and Lopes, 2020; Kouri et al., 2020; Schulz et al., 2020; Engwall et al., 2021; Halbach et al., 2021; Leeuwestein et al., 2021; Cumbal, 2022; Konijn et al., 2022). A striking result is that only one of these studies used students’ L1 to support learning of the L2 (Leeuwestein et al., 2021). All other studies did not include students’ L1. Also, no studies on educational robots using students’ L1 in other subjects than language learning were found.

## Discussion

The aim of this mini review was to find out how languages are employed in RALL to support learning, and to identify opportunities to use robots within a translanguaging pedagogy. A total of 83 articles on RALL were analyzed on their usage of L1 and L2. Most studies focused on using either the L1 or the L2 as the main language of communication, with occasional use of the other language. Few studies so far used a mix of L1 and L2 in student-robot interaction, and only one study used the L1 of students with a migrant background. L1s have rarely been used to support students’ (language) learning, and effective translanguaging exercises (such as discussing assignments within class in the L1 or pre-teaching in the L1) have not been observed so far.

Perhaps this is due to the notion of translanguaging appearing somewhat counterintuitive upon first sight: more use of the L1 encourages learning of the L2. Although benefits of multilingualism have been established in psycholinguistic research for quite some time, it has proven difficult to incorporate such approaches in education. The lack of translanguaging strategies could also be due to practical concerns. The high heterogeneity of migrant populations makes it challenging to adapt to each student’s L1. It is easier, of course, to design student-robot interactions in one language for all participants. However, I would like to invite all researchers to embrace the challenge of designing multilingual interactions for students of different backgrounds. It is especially useful for multilingual students to use their L1s in education, and robots can then be used in a way that they are truly an addition to current classroom practices, as they can do something that most teachers cannot, namely, speaking all multilingual children’s home languages.

This review shows that there are ample opportunities for new research in the field or RA(L)L. It could be advantageous for many students–both monolingual and bilingual–if more researchers set out to investigate the use of robots in a translanguaging pedagogy. Many teachers do not speak children’s L1s, and robots can therefore fulfill a unique role for migrant children within school environments. Perhaps they can pre-teach children in the L1, provide translations in the L1 for difficult vocabulary, or be the conversation partner with which assignments can be discussed in the L1. Such activities need not be limited to language learning; translanguaging is a valuable approach in learning any aspect of the curriculum. For example, such approaches can also be used in mathematics education, for which children may have obtained previous knowledge in the L1 rather than the L2 (such as count words or concepts such as ‘more’ or ‘adding’). The recent example of a robot acting as a facilitator for positive interactions between children of different language backgrounds (Kim et al., 2021) could be extended to educational situations in which all children’s L1s are positively valued and actively used in learning of any subject.

A limitation of this review is that for some articles, the use of languages was not described in detail. Many researchers described to use only the L2 in student-robot interactions, but did not describe language use amongst students or with the teacher. Perhaps teachers or classmates provided support in the L1. Moreover, a rationale behind the use of languages was often not presented, making it unclear whether the choice to (almost) exclusively use the L1 or the L2 is a strategic choice from a language-learning perspective, or a practical choice from a design perspective. The balance between L1 and L2 in a language-learning activity affects the outcomes of a RALL study and should be carefully considered when designing an experiment.

In conclusion, the main outcome of this review is that multilingual students’ L1s have rarely been used to strategically support learning of the L2: interactions rely heavy on either the L1 or the L2, depending on the context and on students’ previous knowledge. Little use of L1s has been observed especially for students with a migrant background. There are clear opportunities for further research in RA(L)L: robots can be used to facilitate the use of students’ L1s both for language learning and learning in general. This review aimed to provide inspiration for a new line of research in RA(L)L, in which robots can support the active use and positive valuation of any language that students speak.

## Footnotes

1 RALL reviews that were cross-referenced for additional articles were Kanero et al. (2018b), Randall (2019), van den Berghe et al. (2019), Neumann (2020), Lee and Lee (2022), and Lin et al. (2022).
